# Optimized Design of Lithium Niobate Tuning Forks for the Measurement of Fluid Characteristic Parameters

**DOI:** 10.3390/mi14122138

**Published:** 2023-11-22

**Authors:** Man Tang, Dehua Chen, Mi Zhang, Feng Jiang, Yu Wang

**Affiliations:** 1 State Key Laboratory of Acoustics, Institute of Acoustics, Chinese Academy of Sciences, Beijing 100190, China; tangman21@mails.ucas.ac.cn (M.T.); jiangfeng21@mail.ioa.ac.cn (F.J.); wangyu2021@mail.ioa.ac.cn (Y.W.); 2 University of Chinese Academy of Sciences, Beijing 100049, China; 3 Beijing Engineering Research Center of Sea Deep Drilling and Exploration, Institute of Acoustics, Chinese Academy of Sciences, Beijing 100190, China; 4 School of Intelligent Technology and Engineering, Chongqing University of Science and Technology, Chongqing 401331, China; 2021052@cqust.edu.cn

**Keywords:** lithium niobate, tuning fork, dimension optimization, quality factor

## Abstract

The unique double-cantilever beam structure and vibration mode of the tuning fork enable the measuring of fluid density and viscosity synchronously in a decoupling manner. Therefore, it is widely employed in oil and gas development and in petrochemical, food, textile, and other industries. In this paper, quality factors are used to characterize the energy losses of lithium niobate tuning forks when vibrating in a fluid, and the influence parameters, such as length, width, and thickness of the tuning fork arm, etc., of different quality factors are examined with a focus on the viscous quality factor of the fluid. The optimized design of lithium niobate tuning fork dimensions is carried out on this premise, and the analytical solution of the optimal dimension of the lithium niobate tuning fork in the air is obtained. Secondly, the optimal dimension of the lithium niobate tuning fork in fluids is given out by finite element simulation, and the sensitivity of the optimized fork to the viscosity of fluids is investigated. The results show that the optimized tuning fork has a higher quality factor, and thus has a larger parameter measurement range as well as being more sensitive to the change in the fluid density and viscosity. Therefore, the results are of great significance for guiding the preparation and practical application of lithium niobate tuning forks.

## 1. Introduction

Measuring the density and viscosity of fluid is widely needed in the industrial field and useful for guiding production steps. For example, in the production or synthesis of various chemicals, monitoring the change of their density and viscosity under high temperature and pressure is very important because the changing trend can greatly affect the quality of the chemicals [1]. Moreover, the measurement is also useful in other fields, including transformer oil quality monitoring [2], the start-up vibration characterization of space shuttles [3], wine brewing monitoring, bioconcentration detection in the biomanufacturing business [4], and so on.

The tuning fork (TF) sensor can achieve simultaneous decoupled measurement of density and viscosity due to its unique double-cantilever beam structure and vibration mode, and it is simple to produce, lower cost, and easier to implement. Quartz tuning forks (QTFs) were researched earliest and are widely used in various applications. QTFs are widely used in high-resolution mechanical sensors [5], vibration amplitude sensors of a vibrating magnetic sample [6], gas sensors [7], and other areas. The QTF is also commonly used in measurements of fluid parameters because of its high quality and simplicity in use [8].

The Curie temperature of lithium niobate (LN) crystals is 1210 °C compared to 575 °C for quartz crystals [8]. This may suggest that at high temperatures, LN crystals are more stable than quartz crystals. In terms of piezoelectric strain constants, most quartz tuning forks utilize $d_{21}$, which does not exceed $6\times 10^{-12}\;C/N$, while lithium niobate forks utilize $d_{23}$, which can reach $28\times 10^{-12}\;C/N$ [9]. Quartz crystals have a smaller piezoelectric strain constant relatively and may not always coordinate with the standards of high sensitivity and high accuracy applications. The electromechanical coupling coefficient of LN crystals is as high as 0.68, compared to a maximum of 0.3 for quartz crystals [10]. Lithium niobate (LN) crystal, with a high electromechanical coupling coefficient and high temperature and pressure resistance, has become a popular material for piezoelectric transducers and sensors in recent years [11]. The TF made of LN has also developed rapidly in recent years, and its excellent characteristics, such as high temperature and pressure resistance, make it expected to become a new direction in the development of well fluid sensors. Jintai Chen et al. used XY-cut LN as a substrate for temperature-sensitive surface acoustic wave resonators, as it shows stable physical and chemical properties over a wide temperature range [12]. Toda et al. investigated the effect of oil density and viscosity on the cantilever beam of lithium niobate tuning forks with embedded electrodes, and the forks’ size was 6.3 mm × 1.2 mm × 0.5 mm [13]. Aoust et al. derived the optimal quality factor for tuning forks in fluid media, mainly analyzing the optimal design of the dimensions of quartz tuning forks [14]. Gonzalez tried to use lithium niobate tuning forks for measuring the density and viscosity of downhole fluid rapidly, and the sensor had a size of 8 mm × 1 mm × 1 mm [15]. Turutin presented a composite tuning fork sensor made of 128° Y-cut lithium niobate crystals and metals, which had a highly magnetic susceptibility but with less description of the dimensional design principles [16].

Lithium niobate tuning forks (LNTFs) are a good choice for measuring the density and viscosity of downhole fluid. However, LNTFs appear very rarely on the market and most articles on LNTFs use the same dimension. It is necessary to optimize the dimensions of LNTFs with a high-quality factor in the fluid, thus achieving a wider parameter measuring range so that they can be used in a wide range of applications. Nevertheless, the selection of LNTF dimensions and optimization concerns have received less attention in the study indicated above. This paper focuses on the optimal design analysis of the dimensions of LN sensors based on energy loss theory, selecting several optimized LN sensors for different application scenarios to analyze their sensitivity to liquid density and viscosity.

## 2. Materials and Methods

In this section, the vibrating model of LNTFs in fluid is constructed, and the coupling law of the first-order reverse flexural in-plane mode of the fork’s vibration to fluid density and viscosity is studied. According to Euler–Bernoulli’s theory, the flexural vibration equation of a cantilever beam equation is solved and the different quality factors of the LNTF are analyzed according to energy loss theory.

### 2.1. The Resonant Frequency of LNTF

In this study, the LNTF is considered to be vibrating in an endless, compressible fluid because the amplitude of its vibration is significantly smaller than the fork’s geometry.

**Figure 1:**
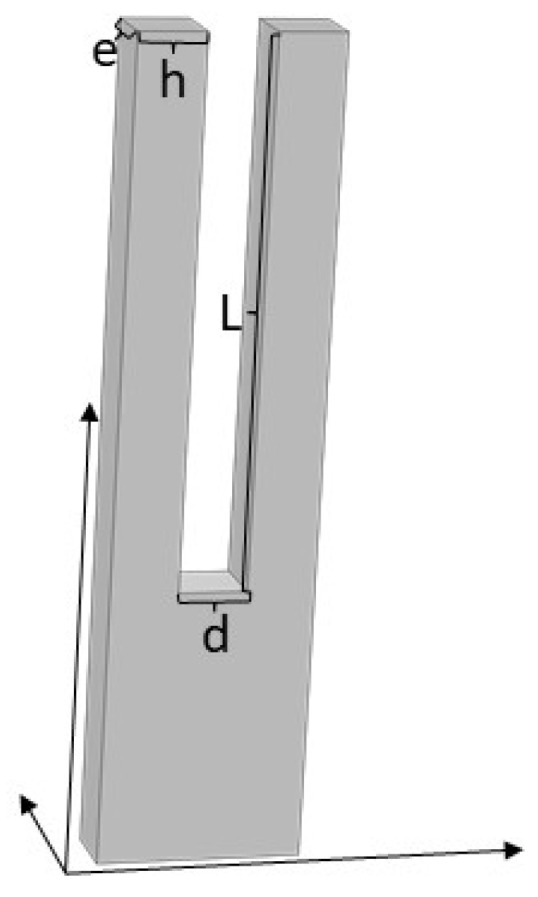
Lithium niobate tuning fork model.

Figure 1 depicts a model of a LNTF. The arm’s length *L* is significantly greater than its width *h* and thickness *e*, thus it can be compared to the vibration of a cantilever beam with one end fixed and the other end free, whose resonant angular frequency in a vacuum is [17]

(1) $$\omega_{vac}=\frac{h}{2\sqrt{3}}\sqrt{\frac{E}{\rho_{g}}}{\left(\frac{1.875}{L}\right)}^{3}$$

where *E* and $\rho_{g}$ are the Young’s modulus and density of the arm’s material, respectively.

The LNTF’s resonant angular frequency of the first-order reverse flexural vibration in fluid is related to $\omega_{vac}$ as [18]

(2) $$\omega_{vac}^{2}=\omega_{1}^{2}(1+\frac{\pi h}{4\rho_{g}e}\rho (1+4\sqrt{\frac{2\mu }{\rho \omega_{1}e^{2}}}(1+\frac{h}{\pi e})))$$

where $\omega_{1}$ is the angular resonant frequency in the fluid [19], ρ is the fluid’s density, and μ is the viscosity of the liquid.

### 2.2. The Quality Factors of LNTF

The dimensions of the LNTF can be optimized based on the energy loss as being represented by the reciprocal of the quality factor; the greater the quality factor, the less energy is lost. The total energy dissipation of the LNTF in the fluid is $\frac{1}{Q_{tot}}$. Material support loss $\frac{1}{Q_{s}}$, fluid viscosity loss $\frac{1}{Q_{v}}$, and acoustic radiation loss $\frac{1}{Q_{a}}$ are the three basic factors that contribute to the total energy dissipation [18].

(3) $$\frac{1}{Q_{tot}}=\frac{1}{Q_{s}}+\frac{1}{Q_{v}}+\frac{1}{Q_{a}}$$

Lithium niobate crystals cut in the 128°Y direction are used in this work. The parameters used in the calculation are $\rho_{g}=4700\;{\mathrm{kg}/m}^{3}$ and $E=1.45\times 10^{11}\;\mathrm{Pa}$, and ν=0.25 is the Poisson ratio [13].

The three-part energy loss’s expression is displayed below:

(4) $$\frac{1}{Q_{s}}={\left(\beta_{n}\chi_{n}\right)}^{2}\frac{(1+\nu )\psi }{0.24(1-\nu )}{\left(\frac{h}{L}\right)}^{3},$$

(5) 1Qv=2(1+hπe)2Re+2Re4ρghπρe+1+2(1+hπe)2Re,

(6) $$\frac{1}{Q_{a}}=\frac{\rho e}{2\rho_{g}}\sum_{m=0}^{m=+\infty }\frac{2}{1+\delta_{0,m}}{\left[J_{2m}(k(h+\frac{2}{d}))-J_{2m}(\frac{kd}{2})\right]}^{2},$$

where ψ is a constant related to the arm material, $\beta_{n}$ and $\chi_{n}$ are the order number of vibrational modes, Re is the Reynolds number, $J_{2m}$ is a first-order Bessel function, *k* is the wave number, and $\delta_{0,m}$ is the Kronecker symbol. In this paper, the first-order reverse flexural in-plane vibrational mode of LNTFs is considered; therefore, *n* = 1, $\beta_{1}=0.597$, and $\chi_{1}=-0.734$ are substituted into the calculation [18].

## 3. Results

### 3.1. Calculation and Analysis of Resonant Frequency and Quality Factors

As the vibration of the LNTF in the air can be approximated as free boundary conditions, all of the above formulae can be solved analytically, and so the dimensions of the LNTF were initially optimized using the case in the air as an example.

A LNTF’s vibration in the air can be approximated as free vibration, and its resonant frequency can be equal to the resonant frequency in a vacuum, i.e., $\omega_{1}=\omega_{vac}$. In this paper, the parameters of air at 25 °C are $\rho =1.169\;{\mathrm{kg}/m}^{3}$ and dynamic viscosity $\mu =18.448\times 10^{-3}\;\mathrm{mPa}\cdot s$. The relationship between resonant frequency, quality factors, and the characteristics of the fork’s dimension can be examined using the theoretical formulas mentioned above.

#### 3.1.1. Resonant Frequency

Equation (1) gives the resonant frequency of the LNTF in the air.

**Figure 2:**
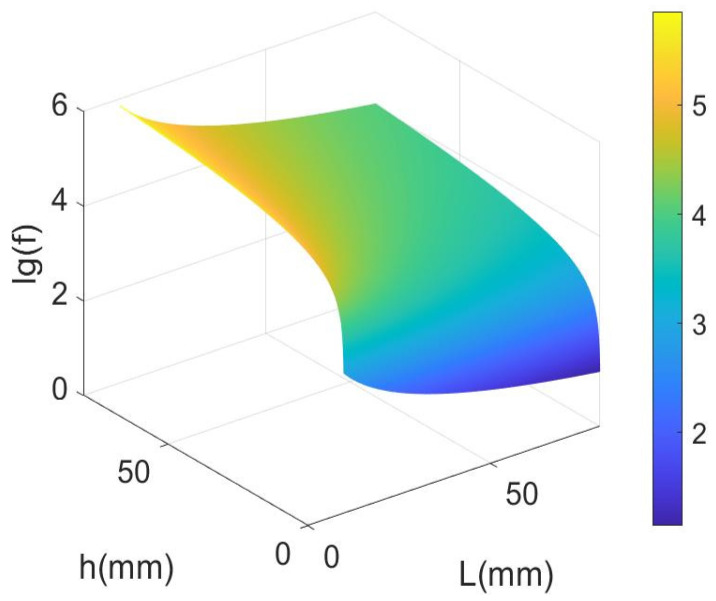
The relationship between the resonant frequency of LNTF and the length and width of its arm.

Figure 2 gives the relationship between the resonant frequency of LNTF and the length and width of its arm (the identity lg on the vertical coordinate of the graph is a log function with base 10 that is monotonically increasing from 0 to ∞). The result shows that the resonant frequency *f* is growing with the increase in *h* while dropping with the increase in *L*. Moreover, *L* is much greater than *e*, thus it has a far greater effect on the resonant frequency than *e*. As a result, the low-frequency measurements of the LNTF require a greater length and a smaller width.

#### 3.1.2. Material Support Quality Factor

**Figure 3:**
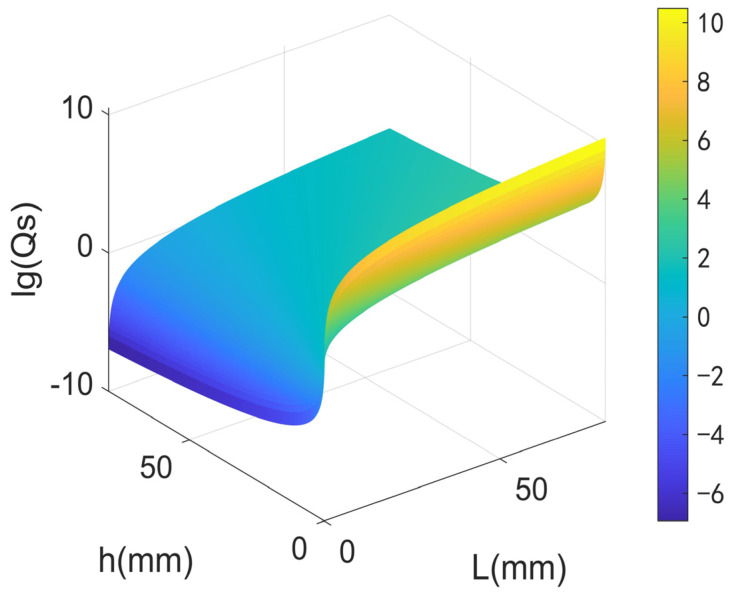
The relationship between *Q_s_* and the length and width of the LNTF’s arm.

Equation (4) demonstrates that the material support loss quality factor $Q_{s}$ is mostly connected to the aspect ratio, thus the relationship between $Q_{s}$ and the length *L* and width *h* of the LNTF’s arm can be calculated as shown in Figure 3.

The result in Figure 3 illustrates that $Q_{s}$ increases with the increase in *L* and decreases with the increase in *h*. In order to obtain a quantitative optimal dimension of LNTF, the relationship between $Q_{s}$ and the ratio of *L* to *h* needs to be further analyzed.

**Figure 4:**
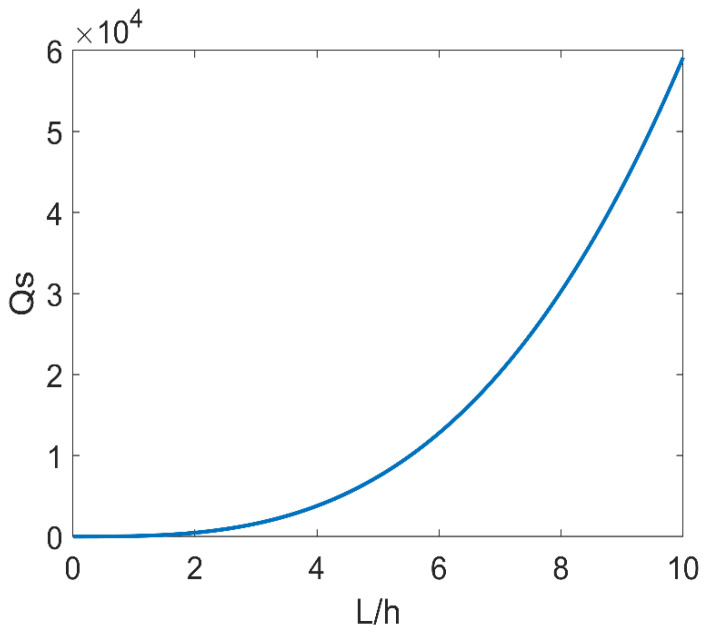
The relationship between *Q_s_* and the ratio of length to width.

Figure 4 shows that $Q_{s}$ gradually increases as the ratio of length to width increases as *L* changes from 0 mm to 80 mm. It demonstrates that the larger the ratio, the lower the material support loss. This law can be applied to the design of LNTFs. Selecting a larger ratio of length to width will result in larger $Q_{s}$ (less material support loss). However, the LNTF’s support stiffness must be considered. The ratio of length to width should not be too large because of the large Young’s modulus of lithium niobate crystals, which might lead to possible fracture even at low frequencies.

#### 3.1.3. Fluid Viscosity Quality Factor

**Figure 5:**
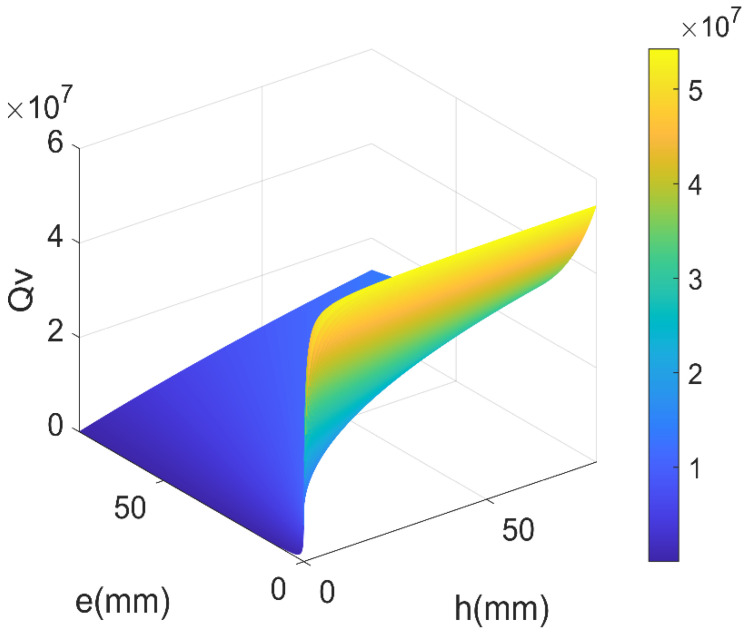
The relationship between *Q_v_* and the width and thickness of the LNTF’s arm.

The fluid viscosity quality factor $Q_{v}$ can be obtained by Equation (5), and it mostly depends on the width *h* and thickness *e* of the LNTF’s arms. Figure 5 shows the relationship between $Q_{v}$ and the width and thickness of the LNTF’s arm at *L* = 10 mm.

It can be seen in Figure 5 that $Q_{v}$ goes up with the width and goes down with the thickness and is affected by thickness largely. So as to obtain higher $Q_{v}$, the impact of the width-to-thickness ratio needs to be further examined.

**Figure 6:**
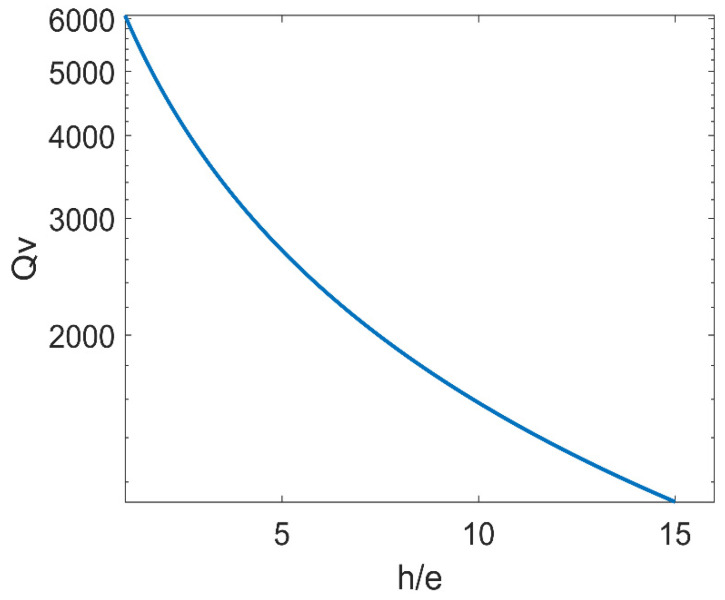
The relationship between *Q_v_* and the width-to-thickness ratio *h*/*e*.

$Q_{v}$ and the width-to-thickness ratio *h*/*e* are related in Figure 6. It is observed that the fluid viscosity loss quality factor $Q_{v}$ falls with the width-to-thickness ratio increasing. Consequently, the thickness should be as large as feasible when the width is certain. This will enhance $Q_{v}$ and increases the LNTFs’ measuring range for fluid viscosity.

#### 3.1.4. Acoustic Radiation Quality Factor

**Figure 7:**
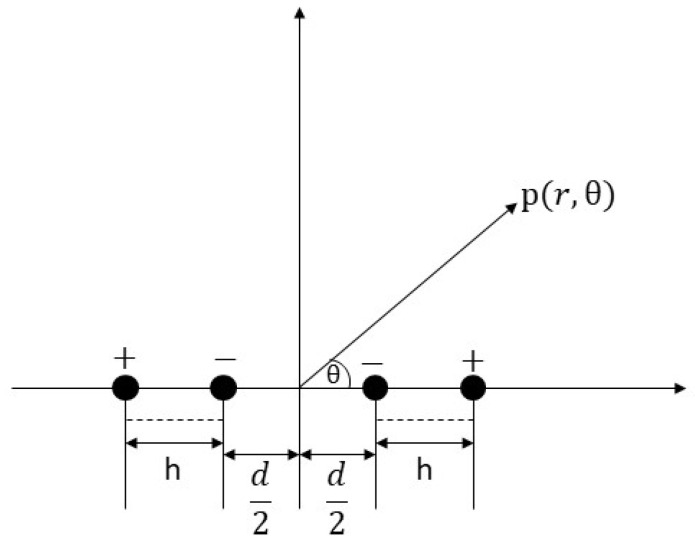
Equivalent point source model of LNTF.

For determining the acoustic radiation parameters, the first-order inverse flexural vibration of the LNTF can be approximated as a longitudinal quadrupole source. Therefore, the acoustic radiation quality factor of the source can be described by the factor of a longitudinal quadrupole source. Figure 7 depicts the comparable two-dimensional acoustic model [20].

According to Equation (6), the acoustic radiation quality factor $Q_{a}$ is proportional to the width *h* and thickness *e* of the LNTF’s arm, as well as the fork finger spacing *d*.

**Figure 8:**
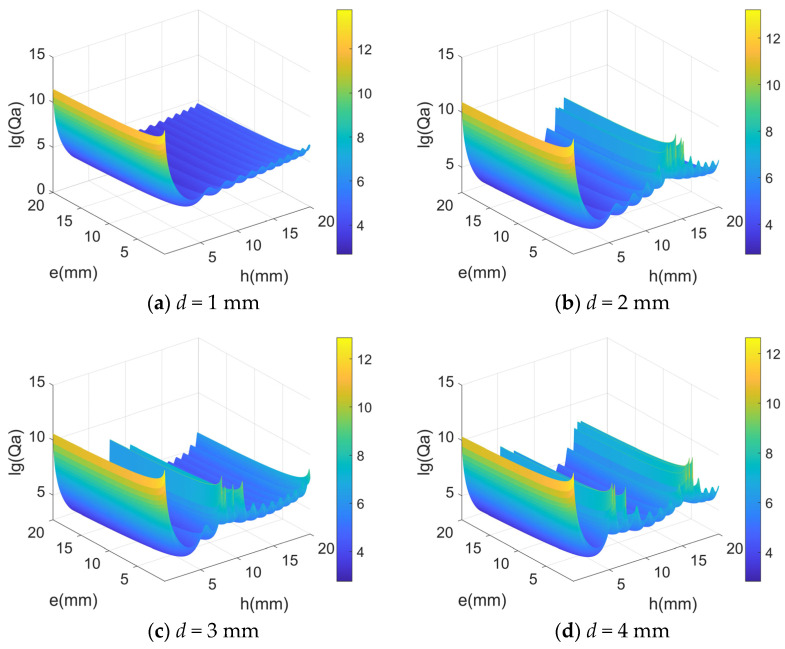
The relationship between *Q_a_* and width *h*, thickness *e*, and the spacing *d*.

Figure 8 gives the relationship between $Q_{a}$ and width *h*, thickness *e*, and the spacing *d*. The results show that $Q_{a}$ is almost insensitive to the spacing *d* at small width and thickness *e* and decreases sharply with the increasing of width and gradually with the increasing of thickness. Therefore, a small width and thickness *e* should be selected to maximize $Q_{a}$.

#### 3.1.5. The Total Quality Factor

**Figure 9:**
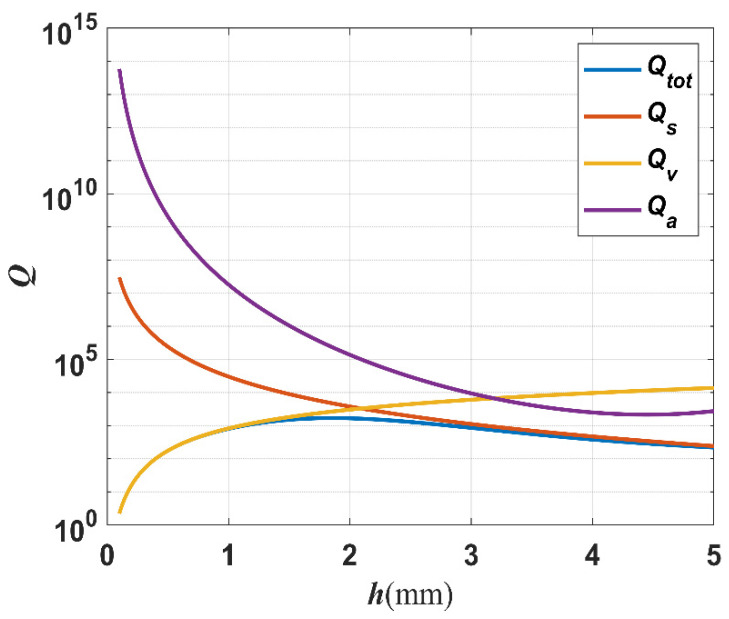
The relationship between different quality factors and the width of the LNTFs’ arm.

The total quality factor $Q_{tot}$ can be obtained from Equation (3). Taking a certain length of 10 mm and a width-to-thickness ratio of 1 as an example, other quality factors with the trend of change in width are shown in Figure 9.

As seen in Figure 9, the total quality factor $Q_{tot}$ is dominated by the fluid viscosity quality factor $Q_{v}$ in regions that are sensitive to fluid viscosity when the *L* = 10 mm, the width-to-thickness ratio is 1, and the width is less than 2 mm. With the width larger than 2 mm, $Q_{tot}$ depends mainly on the material support loss quality factor $Q_{s}$ and is relatively lower, which causes the LNTF to be less sensitive to fluid viscosity and is unsuitable for measuring high-viscosity fluids.

#### 3.1.6. Analysis of Theoretical Results

Combining the previously mentioned theoretical calculations, the rules of the optimized design of a LNTF in air are as follows:

1. The larger the length-to-width ratio, the higher the material support quality factor;
2. The longer the length, the smaller the width, and the lower the resonant frequency;
3. The smaller the width-to-thickness ratio, the greater the fluid viscosity quality factor when width *h* is determined;
4. The acoustic radiation quality factor is greater when both width and thickness are as minimal as possible.

The total quality factor rises firstly and then falls as the width grows when the length *L* is 10 mm, the width-to-thickness ratio is 1, and the fork finger spacing is 1 mm. The total quality factor with the rising trend is primarily related to the fluid viscous loss quality factor, and choosing a TF’s arm width within this range can make it more sensitive to air viscosity.

### 3.2. Finite Element Numerical Simulation

Due to the complexity of the boundary conditions when the LNTF is in a fluid, a different method is needed to obtain the analytical solution of its resonant frequency and quality factor. Therefore, the finite element method is used to analyze the dimension optimization of LNTFs in fluids.

Several dimensions of LNTF are chosen in conjunction with the application scenarios, the resonant frequency and quality factor are calculated using the finite element method in COMSOL5.6 software, and the LNTF dimension is optimized based on the simulated results.

#### 3.2.1. The Principle of Designing the Dimension of LNTF

By optimizing the design of LNTFs in air, the thoughts can be obtained as follows: after defining the length of the LNTF arm, the width can be determined to enable a low resonant frequency and a high support loss quality factor of the material; once the LNTF arm’s width has been set, a thickness can be identified so that the fork has a higher quality factor for both fluid loss and sound radiation. These principles can be utilized to determine the ideal length, width, and thickness in various application settings to maximize energy loss reduction and enhance the LNTF sensor’s quality factor.

#### 3.2.2. Finite Element Simulation

The length must not be set too long in order to meet the requirements for application in the borehole, even though the total quality factor grows with the length. Although the quality factor can take a larger value as the length increases, the length-to-width ratio also gradually increases. Eventually, combined with the Young’s modulus analysis of lithium niobate material, the length should not be too large.

In this work, *L* = 10 mm and *L* = 8 mm are chosen for analysis. Choosing a width-to-thickness ratio of 1 and the fork finger spacing *d* of 1 mm, based on the results in Figure 9, the LNTF arm’s width *h* can be selected in the range from 0.8 mm to 2 mm with a step of 0.2 mm.

Table 1 shows the analytical results and numerical simulating results of various LNTFs’ resonant frequencies in vacuum.

Table 1 demonstrates that as the width of the LNTF’s arm increases, its resonant frequency gradually increases, and the difference between the analytical results with single-cantilever beam theory and the finite element simulating results also increases.

**Figure 10:**
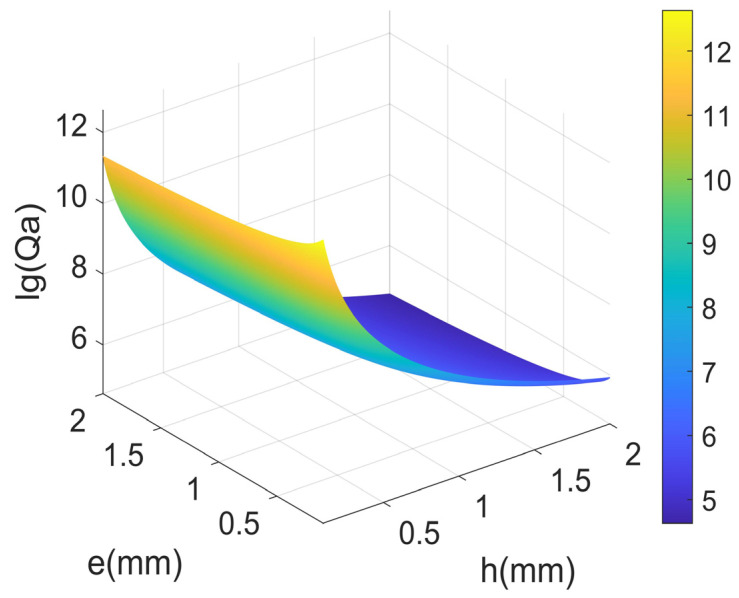
The relationship between the acoustic radiation quality factor in the air and its thickness and width.

From Figure 10, it can be seen that as the width and thickness increase, its acoustic radiation quality factor decreases (more and more energy loss), which may be the reason for the decrease in the resonance frequency of the double-cantilever beams. As shown in Figure 8, the distance *d* also has some minor effects on the acoustic radiation quality factor. By combining the results above, it suggests that the resonance frequency of the tuning fork is also affected by the distance *d*. If the LNTFs’ resonant frequency is limited absolutely, the appropriate result can be obtained by adjusting the distance *d*.

The several fluids involved in the simulations are taken from Refs. [13,21]. Table 2 shows the density and viscosity of each fluid.

First, the influence of density on the LNTF’s resonant frequency is investigated. The most dense fluid, No. 8, is chosen as a reference and the changes in the other fluids are compared to this value.

**Figure 11:**
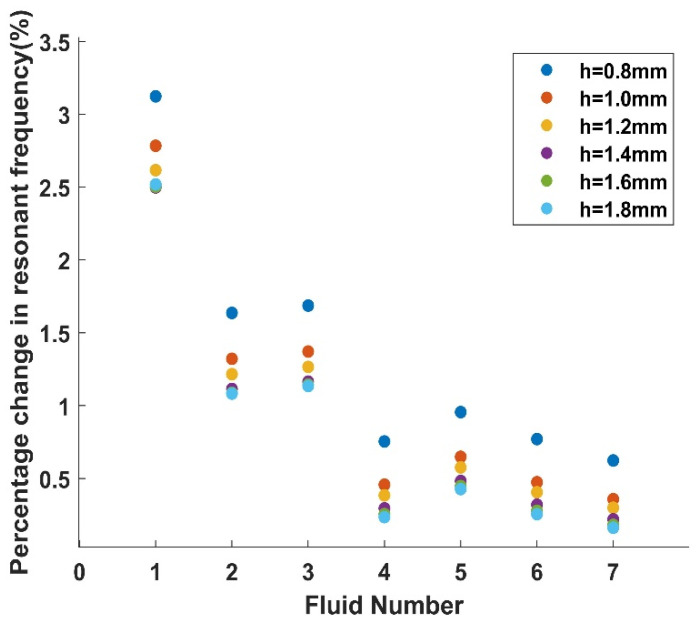
Percentage changes in resonant frequency under different fluids.

As shown in Figure 11, the corresponding variations in the resonant frequencies of various other fluids measured by several LNTFs do not differ significantly from fluid No. 8 with density $861.1\;{\mathrm{kg}/m}^{3}$; this implies that the sensitivity of LNTFs of various widths to variations in density does not vary significantly. These LNTF measurements were found to follow similar tendencies, so it is assumed that all of these LNTFs can predict the density more consistently and precisely.

According to previous research [18], viscosity is the primary component that influences a LNTF’s quality factor, and the higher the viscosity, the lower the quality factor. Since fluid No. 8 has the highest viscosity, its quality factor should be the lowest. This fluid is chosen as a basis for observing the variation in quality factor acquired in the other seven fluids on this basis, as well as the different tendencies of several LNTFs in this situation.

**Figure 12:**
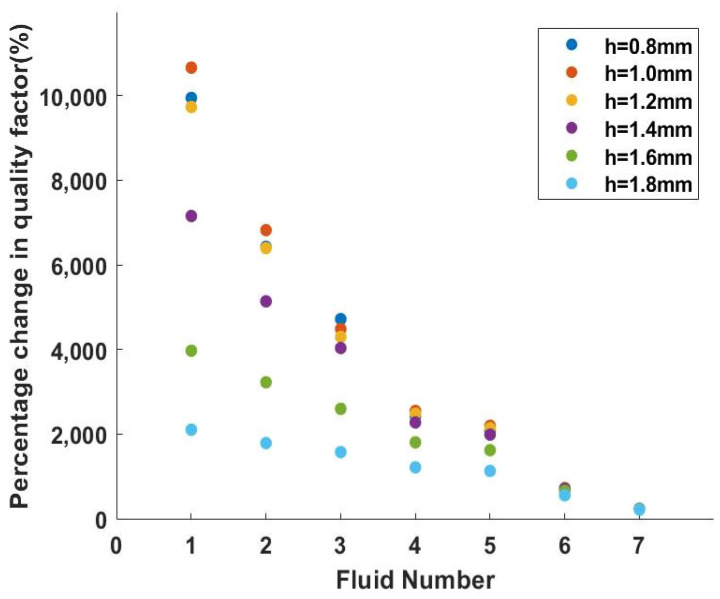
Percentage change in quality factor corresponding to different fluids.

The total quality factor $Q_{tot}$ has the greatest range of change with viscosity at *h* = *e* = 1.8 mm, which is consistent with what we saw in Figure 9. However, Figure 12 shows that the relative percentage change in the total quality factor $Q_{tot}$ is greatest when *L* = 8 mm and *h* = *e* = 1.0 mm, which indicates that it is most sensitive to the changes in viscosity. As a result, the sensitivity of each LNTF to variations in viscosity at high viscosities is examined further below.

**Figure 13:**
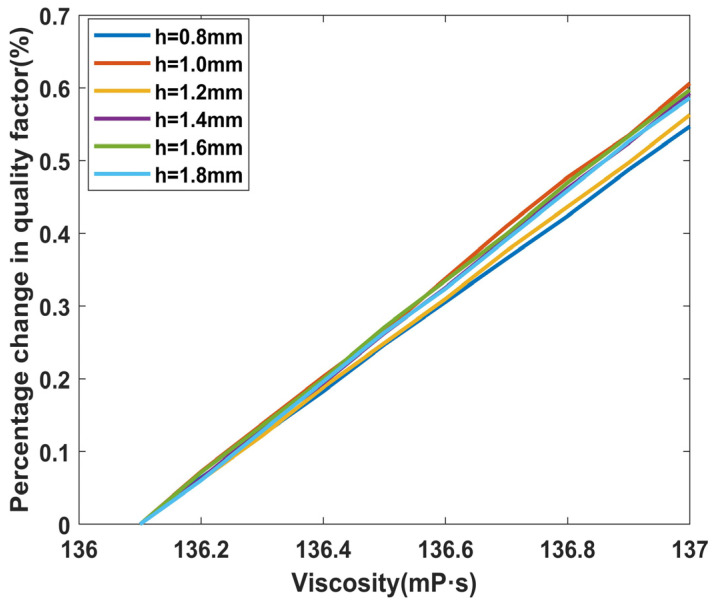
Sensitivity changes of each LNTF under high viscosity.

All of the calculations in Figure 13 use the No. 8 high viscosity fluid as their foundation. Each time the viscosity was increased by 0.1 mPa·s with the density remaining constant, the relative change in the total quality factor of each LNTF was investigated. At *h* = *e* = 0.8 mm, the percentage change in the total quality factor is considerable, and the results indicate that the lithium niobate tuning forks of this dimension are more responsive to viscosity changes at high viscosities, which is consistent with the results in Figure 12.

Combining Figure 12 and Figure 13, the following conclusions can be drawn: the tuning fork with *L* = 8 mm and *h* = *e* = 1.8 mm can be selected to measure higher viscosity fluids (greater range of quality factor changes); to increase the sensitivity of the tuning fork in high viscosity fluids, *h* = *e* = 1.0 mm can be selected. The six tuning forks with *L* = 10 mm are evaluated in the same way below. In the same vein, the width *h* can be selected as 1.0 mm, 1.2 mm, 1.4 mm, 1.6 mm, 1.8 mm, and 2 mm, respectively, when *d* = 1 mm and the width-to-thickness ratio is 1.

**Figure 14:**
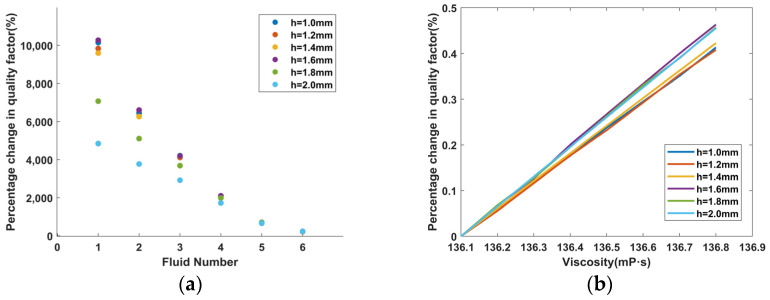
The quality factor of each tuning fork in the fluid when *L* = 10 mm: (**a**) percentage change in quality factor corresponding to different fluids; (**b**) sensitivity changes of each tuning fork under high viscosity.

The fluid No. 8 with the highest viscosity is still selected as the base using the same analytical method. Each tuning fork’s total quality factor in the other fluids in relation to fluid No. 8 is examined. According to Figure 14a, at *L* = 10 mm, the lithium niobate tuning fork has the greatest change in total quality factor at *h* = *e* = 2.0 mm, which is also observed in Figure 9. However, as shown in Figure 14b, at *h* = *e* = 1.6 mm, the relative percentage changes in the total quality factor of LNTF at high viscosity is the largest, indicating that it is the most sensitive to viscosity variations.

Once experimental validations are performed in the future, the above calculations can be utilized as a guidance. In reference [18], the quality factors of three metal tuning forks were compared, and experimental results showed a good match between theory and experiment [18]. Following this, an extensive amount of research on LNTFs needs to be done. In future experiments, our research will also explore the ultimate limits of detection of these devices, just like quartz tuning forks devices [22].

## 4. Discussion

Lithium niobate tuning forks can be used for measuring the density and viscosity of downhole fluid due to their high-temperature resistance. An optimal design analysis related to this kind of TF is needed to achieve a high quality factor for measuring the density and viscosity of different fluids and to meet the demand for a wide parameter measurement range. From the perspective of energy loss, the effect of LNTFs’ dimension on its quality factor and resonant frequency is investigated in this paper. Firstly, the dimension of LNTFs in air is designed based on the single-cantilever beam theory. The analytical results reveal that the longer the LNTFs’ length and the wider its width, the greater its material support loss quality factor. The resonant frequency is mostly determined by the length of the LNTFs’ arm. Smaller width and thickness *e* produces higher LNTFs acoustic radiation quality factors. As the tuning fork length and width-to-thickness ratio are constant, the tuning fork’s total quality factor rises and then falls as its width increases. Under the condition that the TF arm length is 10 mm, the width-to-thickness ratio is 1, and the fork finger spacing is 1 mm, the total quality factor increases and then decreases as the width increases, and choosing a TF’s arm width in the range limited by the increasing total quality factor can make the LNTF more sensitive to the air viscosity.

Based on the results of the above theoretical analysis, the dimension of the LNTF in the fluid is designed by using the finite element method. The results suggested that at a high viscosity range, the LNTF with the arm’s length of 8 mm and the arm width and thickness of 1.0 mm, or with the length of 10 mm and the width and thickness of 1.6 mm, is the most sensitive to fluid viscosity’s changes. The above optimization results can be used in guiding the preparation of LNTFs, facilitating the application of LNTFs for measuring the density and viscosity of downhole fluids, and helping to improve the sensitivity of the tuning forks to highly viscous fluids.

## Figures and Tables

**Table 1:** The resonant frequency of LNTFs with different width *h* as *L* = 8 mm.

Width (mm)	0.8	1	1.2	1.4	1.6	1.8
Theoretical resonant frequency (kHz)	11.214	14.018	16.822	19.625	22.429	25.232
Simulated resonant frequency (kHz)	11.295	13.680	15.904	17.981	19.928	21.745

**Table 2:** Fluid parameters used in the simulation.

Number	Density (kg/m^3^)	Viscosity (mPa·s)
1	679	0.38
2	854	55.52
3	855	36.92
4	848	14.5
5	835	4.74
6	785	1.04
7	781	1.96
8	861.1	136.1

## Data Availability

Data are contained within the article.
